# Transglutaminase 7 Silencing Inhibits Proliferation and Modulates Inflammatory and Apoptotic Markers in Testicular Germ Cell Tumors

**DOI:** 10.32604/or.2025.070104

**Published:** 2025-11-27

**Authors:** Rawabi S. Altuwayjiri, Ibtesam S. Almami

**Affiliations:** Department of Biology, College of Science, Qassim University, Buraydah, 52571, Al-Qassim, Saudi Arabia

**Keywords:** Human malignant, testicular germ cell line, gene expression, knockdown, transglutaminase 7, proliferation

## Abstract

**Objective:**

Testicular germ cell tumors (TGCTs) represent the most common malignancy among young men aged 20–40 years. Transglutaminase 7 (TG7), encoded by *TGM7*, is a poorly characterized enzyme whose function in TGCT remains unknown. This study aimed to assess TG7 expression in clinical specimens and investigate its functional role in a testicular germ cell tumor cell line (NT2/D1).

**Methods:**

TG7 protein expression was evaluated in clinical testicular tissue samples via immunohistochemistry (IHC) and immunofluorescence (IF). Functional analysis was conducted in the NT2/D1 human testicular cancer cell line using Dicer-substrate small interfering RNAs (DsiRNAs) targeting TG7. Gene knockdown efficiency was confirmed by reverse transcription quantitative PCR (qRT-PCR), and protein suppression was validated by immunofluorescence. Cell viability was assessed using the MTT assay. The expression of inflammation and apoptosis-related genes was quantified via qRT-PCR.

**Results:**

TG7 expression was significantly elevated in testicular germ cell tumor tissues, showing approximately a 4.5-fold increase compared to normal testis, with strong localization in tumor nests and stromal compartments. In NT2/D1 cells, TG7 silencing using 20 nM DsiRNA3 led to a dose-dependent reduction in cell viability, with up to 48% inhibition observed at 200 nM (MTT assay, ****p < 0.0001). qRT-PCR analysis revealed significant upregulation of IL6 (3.2-fold), TNFα (2.8-fold), and CASP3 (2.5-fold) mRNA levels following TG7 knockdown (*p* < 0.0001), while p53 expression remained unchanged. These findings support TG7’s role in modulating tumor cell survival, inflammation, and apoptosis via p53-independent pathways.

**Conclusion:**

Collectively, TG7 is significantly overexpressed in TGCT tissues and supports tumor cell viability *in vitro*. This study establishes TG7 as a novel biomarker and therapeutic target in testicular cancer, laying the groundwork for future studies on TG7-targeted interventions.

## 1 Introduction

Testicular cancer is the most common solid malignancy in young males, particularly those aged 20 to 40, and represents a significant health burden worldwide. It accounts for approximately 5% of urological malignancies and 1% of all male cancers [1,2]. This cancer arises within the male reproductive system, often presenting as lumps in the testicles accompanied by pain or swelling in the scrotum. Over 95% of testicular malignancies are classified as testicular germ cell tumors (TGCT), which encompass a histologically diverse group of cancers originating from abnormal fetal gonocytes. These gonocytes fail to differentiate properly into spermatogonia, leading to genetic modifications that progress into germ cell neoplasia *in situ* (GCNIS) and, eventually, invasive TGCT in young adulthood [3,4].

Transglutaminases (TGs; EC 2.3.2.13) are a family of structurally and functionally related enzymes that catalyze calcium-dependent, post-translational modifications of proteins. This is achieved by the formation of covalent bonds between free amine groups (such as protein- or peptide-bound lysine) and the γ-carboxamide groups of glutamines within peptides [5]. TGs are implicated in numerous physiological and pathological processes, including programmed cell death (apoptosis), inflammation, cell adhesion, cell growth, and signal transduction [6–8]. They also play a crucial role in maintaining membrane integrity and act as scaffolds in various cellular contexts. The dysregulation of TG activity, gene expression, or protein levels has been linked to several cancers, such as prostate [9], colorectal [10], epidermal [11], and breast cancers [12]. Members of the TG family, including TG3, tissue-type TGase, and TG6, are known to be expressed in the testes [13]. However, their precise physiological and pathological roles in testicular cancer remain largely unexplored. Despite extensive studies highlighting the involvement of TGs in other cancers, such as TG4 and TG2, in prostate cancer [9,14], TG3 in epidermal cancer [11], and TG2 in colorectal [15,16] and breast cancer progression [17,18]. There is limited data on their gene expression, enzymatic activity, and protein levels in human testicular cancer. This gap in knowledge underscores the need for comprehensive studies to elucidate the molecular characterization of TGs in the context of testicular malignancies.

RNA interference (RNAi) has become a powerful tool in cancer research, enabling the targeted silencing of oncogenes. Small interfering RNAs (siRNAs) degrade specific mRNAs through the RNA-induced silencing complex (RISC), and have shown promise in reducing tumor progression and resistance mechanisms [19]. This technology provides a functional approach to interrogate TG isoform roles in cancer [20,21].

Emerging studies suggest that members of the transglutaminase (TG) family are not only involved in normal tissue remodeling but also contribute to cancer-related processes such as extracellular matrix stabilization, resistance to apoptosis, epithelial-mesenchymal transition (EMT), and immune modulation [18,22]. TG2 has been particularly well-studied in ovarian and pancreatic cancers, where its upregulation promotes metastasis and chemoresistance through NF-κB signaling and the maintenance of cancer stem cell phenotypes [23]. TG3 and TG5, although better known for their roles in skin differentiation and barrier formation, have also been implicated in epithelial malignancies. For instance, TG3 plays a role in skin carcinogenesis and epidermal remodeling [24], and TG5 is involved in epidermal homeostasis and has been studied in the context of inherited and autoimmune dermatoses [25].

In contrast, TG7 remains largely uncharacterized in malignancies. Bioinformatic analyses suggest that TG7, encoded by TGM7, harbors nuclear localization signals and unique regulatory motifs that may indicate cell-type-specific functions or noncanonical roles [26]. However, no functional studies have confirmed its role in tumor development or progression. Given the increasing incidence of testicular germ cell tumors (TGCTs) and the limited understanding of TG isoform involvement in testicular cancer biology, elucidating the expression and function of TG7 may provide novel insights into tumor pathogenesis and therapeutic targeting.

In this study, we used Dicer-substrate small interfering RNAs (DsiRNAs), chemically optimized duplexes that enhanced gene silencing with reduced off-target effects, to knock down TG7 expression [27]. By integrating this approach with phenotypic and molecular assays in a testicular cancer model, we aimed to uncover TG7’s mechanistic contribution to tumorigenesis and evaluate its potential as a novel molecular target in TGCTs.

The present study aims to address these gaps by investigating TG gene expression and protein levels in human testicular cancer. By integrating molecular, biochemical, and protein expression analyses with cutting-edge RNAi techniques, this study seeks to provide novel insights into the role of TGs in testicular cancer pathogenesis. The findings may have significant implications for developing targeted therapeutic strategies, potentially improving patient outcomes with testicular cancer.

## 2 Materials and Methods

### 2.1 Cell Culture of Human Malignant Testicular Germ Cell Line (NT2/D1)

All cell culture procedures were performed under aseptic conditions. The NT2/D1 human malignant testicular germ cell line (embryonal carcinoma) was obtained from the American Type Culture Collection (ATCC, Manassas, VA, USA; Cat. No. CRL-1973). The cell line was authenticated by the supplier using short tandem repeat (STR) profiling, and it was confirmed to be free of mycoplasma contamination. Cells were cultured in Dulbecco’s Modified Eagle Medium (DMEM; ATCC, Manassas, VA, USA) containing high glucose and L-glutamine, and supplemented with 10% (v/v) heat-inactivated fetal bovine serum (FBS, Gibco BRL, Thermo Fisher Scientific, Waltham, MA, USA), 100 U/mL penicillin, and 100 μg/mL streptomycin (Gibco, Life Technologies, Grand Island, NY, USA). Cultures were maintained in T25 cell culture flasks with vented caps (TC-Treated, Biofargo, VA, USA) at 37°C in a humidified atmosphere with 5% CO_2_. Adherent cells were detached using a sterile cell culture scraper (Corning, NY, USA) and collected by centrifugation at. 5000× *g* for 5 min at 25°C [28]. The resulting pellets were resuspended in 1 mL of fresh culture medium. Cell morphology and confluency were routinely monitored under an inverted light microscope (IM-3, OPTIKA, Almenno San Bartolomeo, Italy) at 10× magnification. Cells were observed routinely for morphology and viability under phase-contrast microscopy. Experiments were conducted using NT2/D1 cells at passages 3–6 to ensure consistency in cellular behavior and response.

### 2.2 siRNA-Mediated Knockdown Technique

Short interfering RNAs (siRNAs) targeting TGM7 were designed using Dicer-Substrate siRNAs (DsiRNAs) from the TriFECTa^®^ Kit (CAT#RNA-10025-PR, Integrated DNA Technology, Coralville, IA, USA). Transfection was conducted using the HighGene transfection reagent (CAT#RM09014; ABclonal Technology, Wuhan, China). NT2/D1 cells were cultured in T75 flasks until they reached 70%–90% confluency. The cells were washed with PBS pH 7.2 (1×) (Gibco, Life Technologies, Grand Island, NY, USA), detached mechanically, and transferred into 15 mL conical tubes. After centrifugation at 200× *g*, the cells were counted using a cell counter (Improved Neubauer haemocytometer, Camlab, Cambridge, UK). A density of 0.5–1 × 10^6^ cells/flask or 25,000 cells/well was seeded in either 24- or 96-well plates. Cells were plated in triplicate, with three plates prepared for each experimental condition, and were allowed to grow overnight at 37°C in a humidified incubator with 5% CO_2_.

On the second day, transfection was initiated by adding 500 μL of 20 nM DsiRNA/HighGene complexes in a serum-free medium to the cells. The transfection was performed under the same incubation conditions. Three DsiRNA sequences targeting TGM7 (Table 1) were tested at concentrations of 20 nM. Positive controls (HPRT DsiRNA) (CAT#51-01-08-02: Integrated DNA Technology, Coralville, IA, USA), negative controls (non-targeting siRNA) (CAT# 51-01-14-03: Integrated DNA Technology, Coralville, IA, USA), and untreated cells were included. Transfection efficiency was assessed in some experiments using TYE-563-labeled Transfection Control DsiRNAs at varying concentrations (10, 1, and 0.1 nM). After 24–48 h post-transfection, cells were harvested to evaluate gene knockdown efficiency and examine functional outcomes. These assessments were conducted using quantitative real-time PCR (AriaMx Real-Time (qPCR system); Agilent Technologies, Santa Clara, CA, USA).

**Table 1:** DsiRNA sequences targeting TGM7

CAT# TGM7 DsiRNA	SEQ1	SEQ2
hs.Ri. TGM7.13.1	rGrCrUrUrUrGrUrUrUrArCr ArArGrGrGrUrCrArUrGrArAAG	rCrUrUrUrCrrUrGrACrCrCrUr UrGrUrArArArCrArArArGrCrCrA
hs.Ri. TGM7.13.2	rCrUrArCrArGrCrArArUrUrAr CrArGrArArArCrArArGrCTA	rUrArGrCrUrUrGrUrUrUrCr UrGrUrArArUrUrGrCrUrGrUrArGrGrG
hs.Ri. TGM7.13.3	rGrArArGrCrUrCrUrGrUrUrArGr ArArArCrArCrCrGrUrGTG	rCrArCrArCrGrGrUrGrUrUrUrCr UrArArCrArGrArGrCrUrUrCrArU

Note: The sequence for the non-targeting control siRNA is not disclosed by the supplier (IDT).

### 2.3 RNA Isolation

Total RNA was isolated using the GET™ Total RNA kit (786-132; G-Biosciences, MO, USA). NT2/D1 cells at 80% confluency were homogenized in 200 µL of genomic lysis buffer with a clean pestle, followed by the addition of 5 µL Longlife™ Proteinase K. The mixture was incubated at 60°C for 1 h. After centrifugation at 5000× *g* for 5 min, the supernatant was transferred to a clean tube. To the supernatant, 400 µL of GET Binding Buffer was added. This mixture was loaded onto a GET Silica Column and centrifuged at 12,000× *g* for 1 min at 25°C. The flow-through was discarded, and the column was washed twice with 0.6 mL of GET Washing Buffer, followed by centrifugation at 12,000× *g* for 1 min at 25°C. The column was spun at 14,000× *g* for 3 min to remove residual buffer. It was then placed into a nuclease-free 1.5 mL microfuge tube, and RNA was eluted with 50 µL of 60°C warmed GET Elution Buffer. After a 15-min incubation at 25°C, RNA was collected by centrifugation at 12,000× *g*. Concentration and purity were measured using a NanoDrop ND. 1000 spectrophotometer (Thermo Fisher Scientific, Waltham, MA, USA). Total RNA from normal adult human testis tissue was purchased from Zyagen (CAT#: HT-501, Zyagen, San Diego, CA, USA). This RNA is a pooled sample representing multiple donors and includes RNA from all major testicular cell types.

### 2.4 Quantitative Reverse Transcription Polymerase Chain Reaction (qRT-PCR)

RNA concentration and purity from the NT2/D1 cells were measured using a NanoDrop ND-2000c spectrophotometer (Thermo Fisher Scientific, Waltham, MA, USA). Primers specific to TG1–TG7, CASP3, p53, TNFα, IL6, HPRT, and GAPDH were designed using the PrimerQuest Tool from Integrated DNA Technologies (IDT, Coralville, IA, USA) (Table 2). These primers were optimized to span exon-exon junctions and include all known alternatively spliced mRNA variants.

**Table 2:** Accession numbers and primer sequences for qRT-PCR of TG isoforms and related genes

Gene/Accession number	Forward primer	Reverse primer
**TGM1 NM_000359.3**	5^′^-GATGGCAGCTTCAA GATTGTTT-3^′^	5^′^-GAGCCTTCTGGGTGCT TATAG-3^′^
**TGM2 NM_001323316.2**	5^′^-TGTTGGTCAGAGGAGT GATTG-3^′^	5^′^-GGAGTGGACCTTGTGG TTATT-3^′^
**TGM3 NM_003245.4**	5^′^-TGACGAAGGCTGT GTTTCC-3^′^	5^′^-AGGACTGGAGATGCT GATAGT-3^′^
**TGM4 NM_003241.4**	5^′^-GATGCTGTGGAGCCTT AGTT-3^′^	5^′^-GCTCTTGAATCTGCCCT CATA-3^′^
**TGM5 NM_004245.4**	5^′^-CCTCCAGAGCTCCAGAA ATAATG-3^′^	5^′^-GAAGTACAGGGTGAGGT TGAAG-3^′^
**TGM6 NM_001254734.2**	5^′^-CATCCTGAACATCTG CCTCTC-3^′^	5^′^-TCGGTCGTTGTTGCT GTT-3^′^
**TGM7 NM_052955.3**	5^′^-GGGTCTTCGCCTCTG TTATG-3^′^	5^′^-ATCTCGGCATTTCGGTC ATAG-3^′^
**TNF NM_000594.4**	5^′^-AGAGGGAGAGAAGCAA CTACA-3^′^	5^′^-GGGTCAGTATGTGAGAGG AAGA-3^′^
**IL6 NM_000600.5**	5^′^-GGAGACTTGCCTGG TGAAA-3^′^	5^′^-CTGGCTTGTTCCTCA CTACTC-3^′^
**GAPDH NM_001256799.3**	5^′^-GGTGTGAACCATGGAA GTATGA-3^′^	5^′^-GAGTCCTTCCACGATA CCAAAG-3^′^

The ABScript II One-Step SYBR Green qRT-PCR Kit (Cat# RK20404; ABclonal Technology, Wuhan, China) was used for reverse transcription and qPCR amplification. Three hundred (300 ng) of RNA per sample was reverse transcribed into cDNA according to the manufacturer’s protocol. qRT-PCR was conducted using the AriaMx Real-Time PCR System (Agilent Technologies, Santa Clara, CA, USA). The reaction mixture (20 µL total volume) included 10 µL SYBR Green qRT-PCR buffer, 0.8 µL ABScript II enzyme mix, 0.4 µL forward primer (10 μM), 0.4 µL reverse primer (10 μM), 0.4 µL ROX reference dye (50×), 2 µL RNA, and 6 µL RNase-free water.

Thermocycling conditions included one reverse transcription cycle (5 min at 42°C) and one pre-denaturation cycle (1 min at 95°C), followed by 40 amplification cycles (5 s at 95°C and 30–34 s at 60°C). Melting curve analysis was performed at the end of each run to verify amplification specificity. All reactions were run in duplicate, and the experiments were repeated at least three times for reproducibility. Data was analyzed automatically using the AriaMx software, v2.1.1 (Agilent Technologies, Santa Clara, CA, USA) for comparative quantification. Relative gene expression was calculated using the 2^−ΔΔCT^ method [29] with GAPDH as the internal control. The final results were processed and statistically evaluated using the GraphPad Prism software, v10.2.3 (GraphPad Software, Boston, MA, USA).

### 2.5 Bioinformatics and Software Tools

Primers for qRT-PCR were designed using the PrimerQuest Tool (Integrated DNA Technologies, https://www.idtdna.com/Primerquest/Home/Index; accessed on 05 March 2024). Thermodynamic parameters, including GC content and secondary structure, were verified using OligoAnalyzer 3.1 (https://www.idtdna.com/pages/tools/oligoanalyzer; accessed on 05 March 2024). Amplification data were analyzed using the AriaMx Real-Time PCR Software v2.1.1 (Agilent Technologies, Santa Clara, CA, USA), and statistical significance was assessed using the GraphPad Prism software v10.2.3 (GraphPad Software, Boston, MA, USA).

### 2.6 MTT Assay for Cell Proliferation

To assess NT2/D1 cell viability and proliferation following TG7 knockdown using DsiRNA, the MTT assay (CAT#: E-CK-A341, Elabscience, Houston, TX, USA) was conducted. NT2/D1 cells at 80% confluency were seeded in 96-well plates at a density of 25,000 cells per well [28]. Three plates were prepared with each experimental condition set up in triplicate. Over 48 h, one plate was analyzed daily.

For the assay, 50 µL of 1× MTT working solution (prepared by diluting MTT reagent 1:10 in DMEM) was added to each well, including control wells containing only media. Plates were incubated at 37°C for 2 h, after which the media was discarded and 150 µL of DMSO (Thermo Fisher, Waltham, MA, USA) was added to dissolve the formazan crystals. Absorbance at 570 nm was measured using a microplate reader (Epoch 2 microplate spectrophotometer: Agilent-BioTek, Santa Clara, CA, USA). The results were calculated as the difference between the mean absorbance of control and experimental wells, and the data were analyzed in duplicate across two independent experiments.

### 2.7 Histology and Immunohistochemistry (IHC)

Tissue sections of normal human testis (Code: HuFPT151) and testicular cancer (seminoma) (Code: HuCAT381) were obtained from TissueArray (Derwood, MD, USA). This study was approved by the Institutional Review Board (IRB) of Qassim University, Saudi Arabia (Approval No. 25-37-22). For the use of human tissue sections purchased from TissueArray (Derwood, MD, USA), the supplier confirms that all human tissues were obtained under Health Insurance Portability and Accountability Act (HIPAA)-compliant protocols with informed consent from donors, and in compliance with the Declaration of Helsinki. Slides were deparaffinized, and antigen retrieval was performed using a sodium citrate buffer (pH 6.0) at 100°C for 10 min. Sections were blocked with 0.3% (v/v) hydrogen peroxide for 10 min at room temperature and then incubated in the protein-blocking buffer for 10 min.

Immunohistochemical staining was performed using High-Sensitivity IHC Detection Kit for Mouse and Rabbit Primary Antibody (CAT#abx097195: Abbexa, Cambridge, UK), following the manufacturer’s instructions. Primary antibodies (1:1000, v/v) targeting TG7 (CAT# abx432053: Abbexa, Cambridge, UK) were applied and incubated using an HRP-conjugated polymer detection system. Visualization was achieved using a 0.5% (v/v) DAB chromogen substrate, and tissue sections were counterstained with 0.1% (w/v) hematoxylin. Positive staining was quantified using the ImageJ software (Java 1.8.0: NIH, Bethesda, MD, USA).

### 2.8 Immunofluorescence Staining

Immunofluorescence staining was used to analyze protein expression and localization in tissue slides and NT2/D1 cells. NT2/D1 cells were cultured on 8-well chamber slides (Thermo Fisher Scientific, Waltham, MA, USA), fixed with paraformaldehyde fixation buffer (Elabscience, Houston, TX, USA), and permeabilized using Triton X-100 (Elabscience). Tissue slides were processed similarly, including deparaffinization and rehydration (TissueArray, Derwood, MD, USA). Both samples were blocked with 1% BSA (Sigma-Aldrich, St. Louis, MO, USA) to minimize non-specific binding. Primary antibodies specific to transglutaminase (Abbexa, Cambridge, UK) were applied overnight at 4°C, followed by secondary antibodies conjugated to fluorescent dyes such as Anti-Rabbit IgG-Elab Fluor^®^ 488 or CY5 (Elabscienc). Nuclei were counterstained with DAPI (Thermo Fisher Scientific, Waltham, MA, USA), and the slides were mounted using ProLong^TM^ Gold Antifade Mountant (Thermo Fisher Scientific, Waltham, MA, USA). Fluorescence imaging was performed using an EVOS FL AMF4300 fluorescence microscope (Thermo Fisher Scientific-Fisher Scientific AS, Oslo, Norway) at 20× or 40× magnification. Images were captured to assess protein localization and distribution.

### 2.9 Statistical Analysis

All data were analyzed using the GraphPad Prism software (version 9; GraphPad Software, San Diego, CA, USA). Group comparisons were performed using one-way ANOVA followed, where appropriate, by Tukey or Dunnett’s multiple comparison tests. The results are expressed as mean ± SEM, and the differences were considered statistically significant at *p* < 0.05.

## 3 Results

### 3.1 Transglutaminase (TG) Screening in NT2/D1 Cells Using qRT-PCR

**Figure 1:**
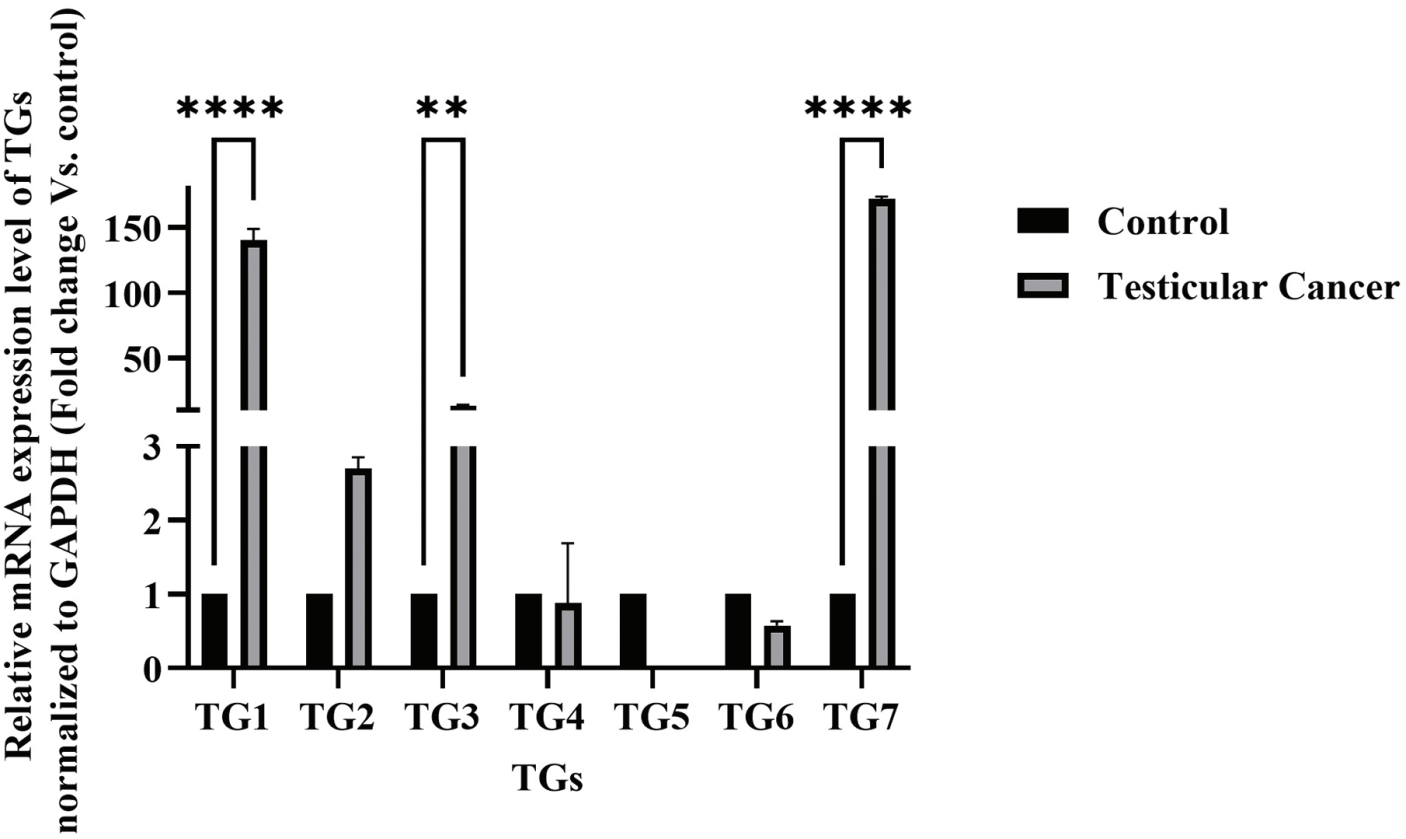
Messenger relative mRNA expression levels of transglutaminases (TG1–TG7) in NT2/D1 cells compared to normal human testicular tissue. Total RNA was extracted from NT2/D1 cells and normal testicular samples, and the expression of TG1, TG2, TG3, TG4, TG5, TG6, and TG7 was analyzed by qRT-PCR using gene-specific primers. GAPDH was used as the internal control. Expression levels in NT2/D1 cells were normalized to those of normal testicular tissue, which was arbitrarily set to 1. The data represent mean ± SEM from three independent experiments (*n* = 3). Statistical analysis was performed using two-way ANOVA followed by Šídák’s multiple comparisons test. **p < 0.01, ****p < 0.0001

To explore the role of transglutaminase in testicular cancer, the gene expression levels of various transglutaminase isoforms (TG1, TG2, TG3, TG4, TG5, TG6, and TG7) were analyzed in the NT2/D1 cell line. As illustrated in Fig. 1, nearly all transglutaminase isoforms are expressed in NT2/D1 cells. Notably, TG7 and TG1 exhibit a highly significant increase in gene expression compared to normal human testes (*n* = 3, ****p < 0.0001). Additionally, TG3 shows a significant increase in gene expression (*n* = 3, **p < 0.01), although to a lesser extent than TG7 and TG1. These findings suggest a potential role for transglutaminase in the progression of testicular cancer.

### 3.2 Immunohistochemical (IHC) Analysis of TG7 Expression in Testicular Tissues

**Figure 2:**
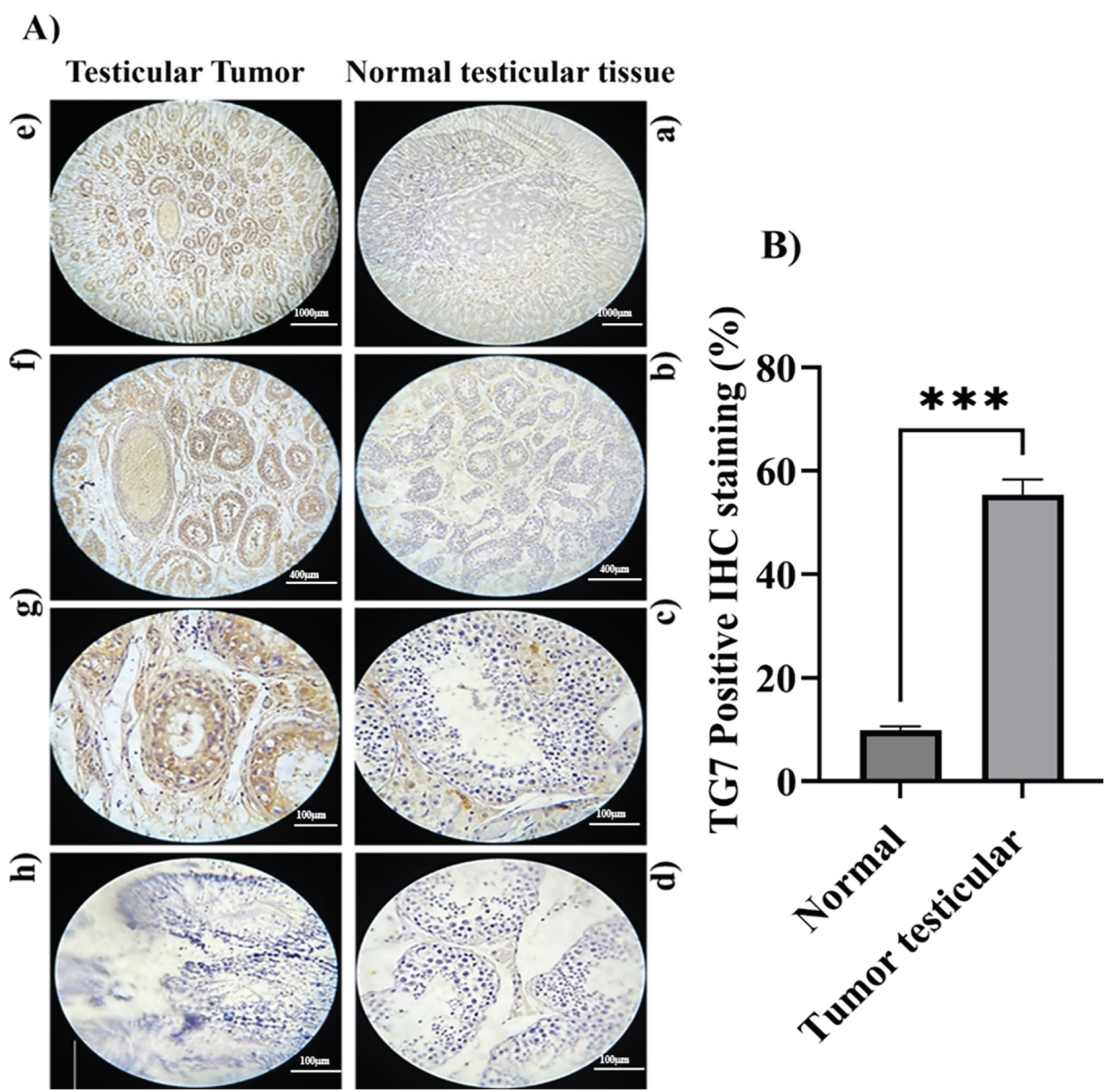
TG7 protein expression and localization in human normal and tumor testicular tissues. (**A**) Immunohistochemical staining of formalin-fixed, paraffin-embedded normal and tumor testicular tissues using a polyclonal anti-TG7 antibody. Panels show representative images at 4× ((**a**,**e**); Scale bar = 1000 µm), 10× ((**b**,**f**); Scale bar = 400 µm), and 40× ((**c**,**g**); Scale bar = 100 µm) magnifications. Negative controls are shown in panel (**b**) for normal and panel (**h**) for tumor tissues (Scale bar = 100 µm). (**B**) Quantification of TG7-positive cells was calculated as a percentage of total cells in each sample. The data represent mean ± SEM from three biological replicates (*n* = 3) per group. Statistical analysis was performed using an unpaired *t*-test; ***p < 0.001

To further confirm the involvement of TG7 in human testicular cancer, the IHC images provide detailed insights into TG7 protein expression, localization, and specific cell types involved. Using immunohistochemical staining with a TG7 polyclonal antibody, differences in TG7 protein levels were observed between healthy and cancerous testis samples (Fig. 2).

The immunohistochemical (IHC) analysis provided detailed insights into TG7 protein expression, localization, and cellular distribution in normal and tumor testicular tissues. In normal tissue (Fig. 2A, panels a–d), TG7 expression is minimal, with weak brown staining sporadically observed in the basal regions of seminiferous tubules, likely corresponding to supporting or germ cells, indicating a negligible role in normal physiology. In contrast, tumor tissues (Fig. 2A, panels e–h) show a marked increase in TG7 staining, with strong and extensive brown staining concentrated in tumor nests and prominently localized within the cytoplasm of tumor cells, particularly those with the large, irregular nuclei indicative of malignancy. Additionally, moderate TG7 staining is evident in the stromal regions adjacent to tumor cells, suggesting potential interactions between tumor cells and the surrounding microenvironment. This pattern of increased TG7 expression, observed in both tumor cells and stromal areas, highlights its potential involvement in tumor cell proliferation, invasion, and stromal remodeling. Quantitative analysis confirmed a statistically significant increase in TG7 expression in tumor tissues compared to normal tissues (*n* = 3, ***p < 0.001). Collectively, these findings suggest TG7 as a critical player in testicular cancer progression, with its strong expression and specific localization supporting its potential as both a biomarker and therapeutic target.

### 3.3 Immunofluorescent Localization of the TG7 Protein in Normal and Testicular Cancer Tissues

**Figure 3:**
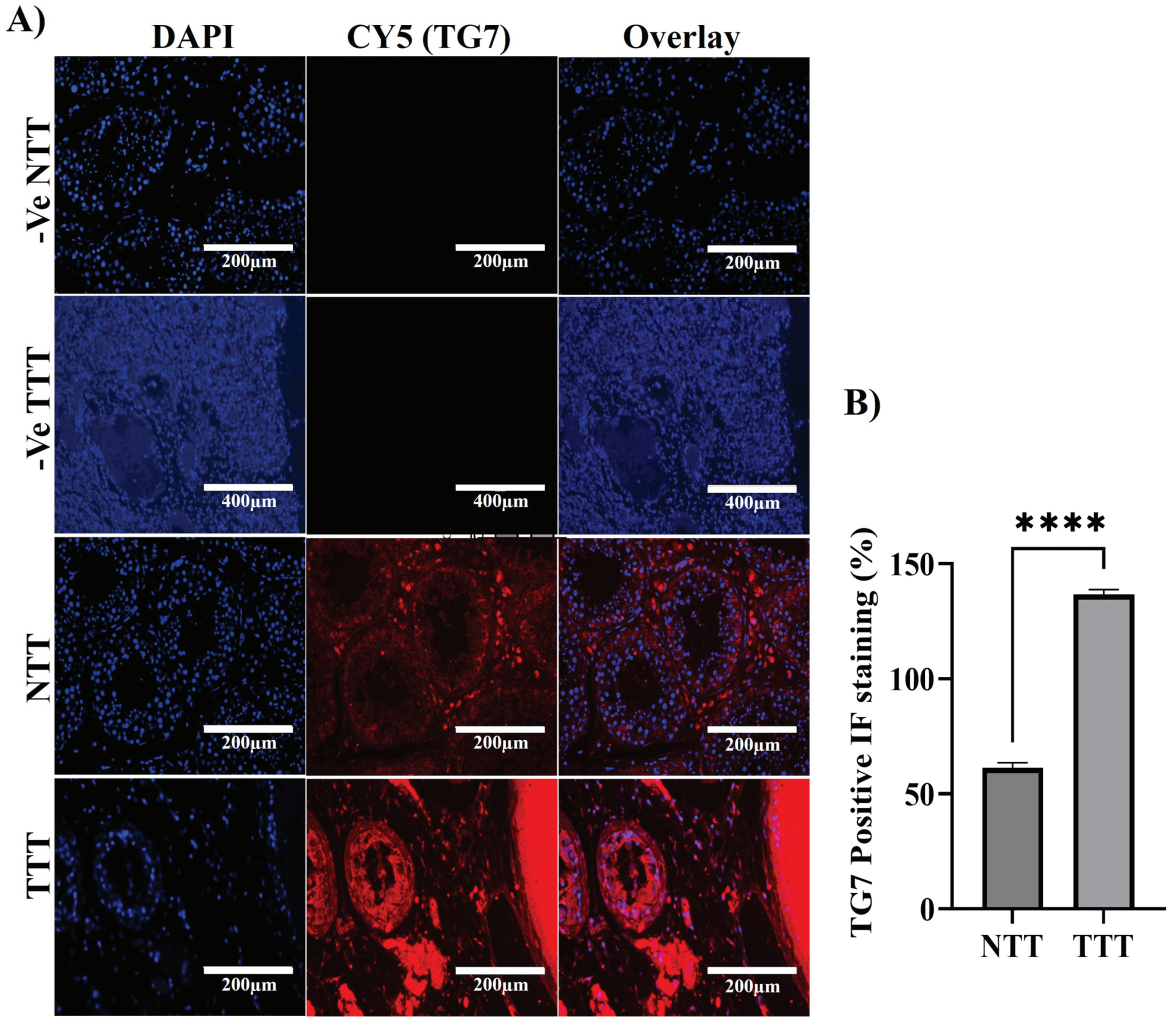
Immunofluorescence analysis of TG7 localization and expression in normal and tumor testicular tissues. (**A**) Immunofluorescent staining was performed on normal testicular tissue (NTT) and tumor testicular tissue (TTT) using a polyclonal antibody against TG7. Negative controls for nonspecific fluorescence are shown for both NTT and TTT (-Ve NTT and -Ve TTT). Images were captured at 10× or 20× magnification; scale bars = 200 µm or 400 µm, respectively. (**B**) Quantification of TG7-positive cells expressed as a percentage of total DAPI-stained nuclei. The data represent mean ± SEM from four independent samples (*n* = 4). Statistical analysis was conducted using an unpaired *t*-test; ****p < 0.0001

To investigate the localization, distribution, and expression levels of TG7 protein in normal human testicular tissue compared to testicular cancer tissue, immunofluorescence (IF) analysis was conducted to better understand TG7’s potential role in testicular cancer development and progression. The analysis reveals distinct differences in TG7 expression between normal and cancerous tissues (Fig. 3). In normal testicular tissue, TG7 protein is predominantly localized between the seminiferous tubules, suggesting its concentration in Leydig cells (interstitial cells of the testes). Within the seminiferous tubules, TG7 expression was relatively low (Fig. 3A).

In contrast, testicular cancer tissue exhibits a significantly higher intensity of TG7-positive red staining throughout the seminiferous tubules, indicating elevated TG7 protein levels. The distribution shifted from being predominantly interstitial in healthy tissue to widespread throughout the seminiferous tubules in cancer tissue. Quantitative analysis of the percentage of positive TG7 IF staining (Fig. 3B) confirmed these observations, showing a statistically significant increase in TG7 expression levels in tumor tissues compared to normal tissues (*n* = 4, ****p < 0.0001).

These findings suggest that TG7 is actively involved in tumorigenic processes, with increased expression correlating with tumor development, which further supports its potential role in the pathophysiology of testicular cancer.

### 3.4 The Effect of the Knockdown of Transglutaminase 7 Gene Expression in a NT2/D1 Human Malignant Testicular Germ Cell Line

#### 3.4.1 siRNA Transfection Efficiency in NT2/D1 Cells

**Figure 4:**
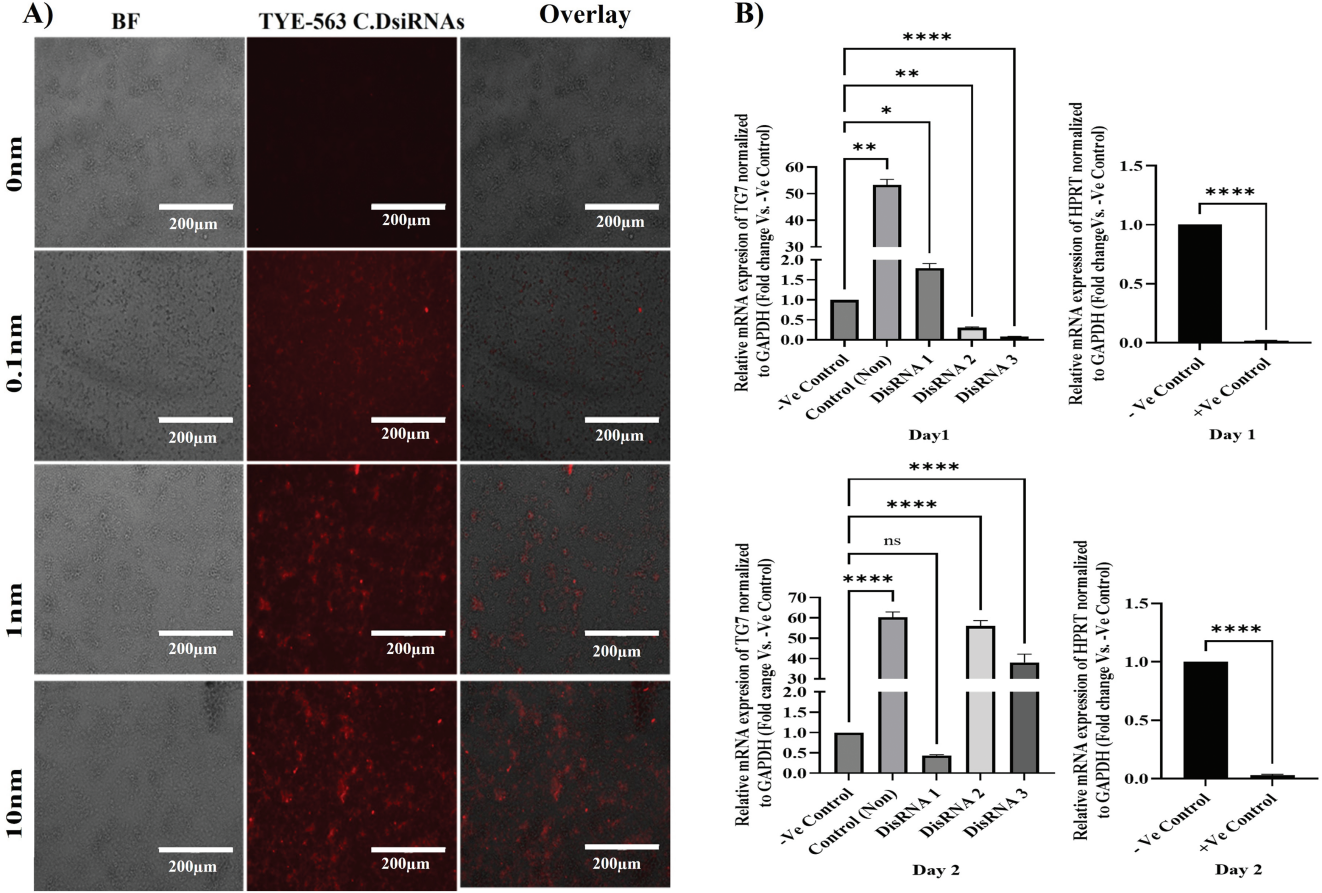
DsiRNA transfection and gene silencing efficiency in NT2/D1 cells. (**A**) Fluorescence and bright-field microscopy of NT2/D1 cells transfected with TYE-563-labeled control DsiRNAs at indicated concentrations (10 nM, 1 nM, 0.1 nM). Scale bar = 200 µm. (**B**) qRT-PCR analysis of TG7 expression in NT2/D1 cells transfected with three different DsiRNAs (DsiRNA1–3) or a positive control targeting HPRT at 24 h (right-upper panel) and 48 h (right-lower panel) post-transfection. Expression was normalized to GAPDH and shown relative to non-targeting siRNA (-Ve control). Data represent mean ± SEM (*n* = 3). Statistical analysis: one-way ANOVA with Dunnett’s multiple comparisons test (TG7 data) and unpaired *t*-test (HPRT data). ns, not significant, *p < 0.05, **p < 0.01, ****p < 0.0001

To study the roles of transglutaminases (TGs) in cancer progression and identify potential therapeutic intervention points, Dicer-substrate short interfering RNAs (DsiRNAs) targeting *TGM7* were designed. The NT2/D1 human malignant testicular germ cell line was transfected using the HighGene transfection reagent. Transfection efficiency was initially assessed using TYE-563-labeled Transfection Control DsiRNAs at varying concentrations (10, 1, and 0.1 nM) of the reagent. As shown in Fig. 4A, 10 nM yielded the highest transfection efficiency, significantly outperforming the lower concentrations (1 and 0.1 nM). These results indicate that 10 nM is the optimal concentration for effective siRNA delivery in NT2/D1 cells.

#### 3.4.2 TGM7 Gene Silencing Efficiency

To evaluate the efficacy of *TGM7* silencing in NT2/D1 cells, three DsiRNA concentrations (20 nM) were tested. Controls included HPRT DsiRNA (positive control), non-targeting siRNA (negative control), and untreated cells. qRT-PCR was used to assess gene knockdown efficiency at 24- and 48-h post-transfection. At 24 h post-transfection (day 1), among the three *TGM7*-targeting DsiRNAs, DsiRNA3 achieved the highest level of gene silencing (*n* = 3, ***p < 0.001), demonstrating the most effective knockdown of *TGM7*. DsiRNA2 exhibited lower knockdown efficiency (*n* = 3, **p < 0.01) compared to DsiRNA3 (Fig. 4B left-upper panel). The *HPRT* DsiRNA positive control confirmed successful transfection and silencing, showing significant knockdown (*n* = 3, ****p < 0.0001) (Fig. 4B, right-upper panel).

The knockdown efficiency pattern shifted at 48 h post-transfection (day 2). DsiRNA1 became the most effective at silencing *TGM7*, surpassing DsiRNA3 and DsiRNA2 (Fig. 4B left-lower panel). The *HPRT* DsiRNA continued to demonstrate significant silencing (*n* = 3, ****p < 0.0001) (Fig. 4B right-lower panel), confirming the sustained effectiveness of the transfection protocol.

The overall knockdown efficacy varied across the two time points, indicating that the silencing kinetics differ among the DsiRNA types. The inclusion of *HPRT* as a positive control validated both the transfection and silencing protocols, ensuring the reliability of the experimental approach. These results highlight the time-dependent variability in DsiRNA efficiency and underscore the potential of DsiRNA1 and DsiRNA3 for targeting *TGM7* in testicular germ cell cancer. The findings provide valuable insights for optimizing siRNA-mediated gene silencing as a targeted cancer therapy.

#### 3.4.3 Validation of TGM7 Silencing Efficiency Immunofluorescence: DsiRNA2 and DsiRNA3 as Optimal Candidates with HPRT as a Positive Control

**Figure 5:**
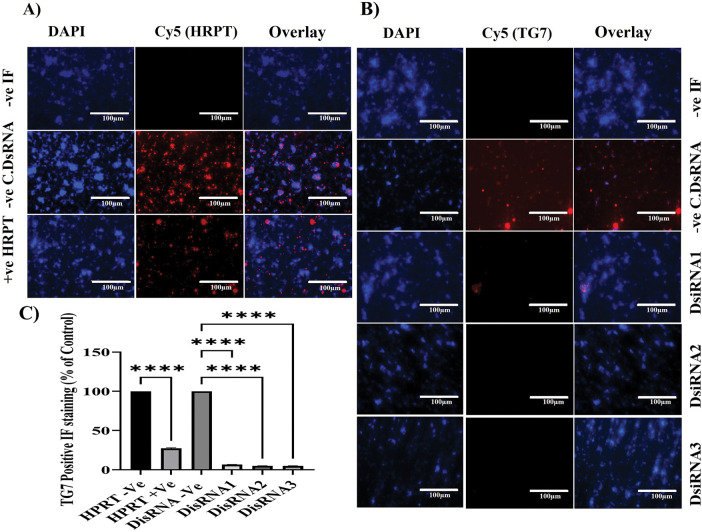
Immunofluorescence analysis of TG7 and HPRT1 protein silencing in NT2/D1 cells. NT2/D1 cells were cultured in chamber slides and transfected with 20 nM of three different DsiRNAs targeting TG7 or the positive control HPRT for 24 h. Immunofluorescent staining was performed using primary antibodies against HPRT (**A**) or TG7 (**B**), followed by Cy5-conjugated anti-rabbit secondary antibody (red). Nuclei were stained with DAPI (blue). Non-transfected cells (-Ve IF) and cells transfected with non-targeting siRNA (-Ve C.DsiRNA) served as the negative controls. Images were captured at 40× magnification; scale bar = 100 µm. (**C**) Quantification of TG7-positive cells as a percentage of total nuclei is shown. The data represent mean ± SEM from three independent experiments (*n* = 3). Statistical analysis was performed using one-way ANOVA followed by Dunnett’s multiple comparisons test; ****p < 0.0001

The initial aim of the immunofluorescence (IF) analysis was to confirm the qRT-PCR findings regarding the silencing efficacy of DsiRNA2 and DsiRNA3 on TGM7 expression in NT2/D1 cells (Fig. 5). HPRT, used as a positive control for DsiRNA efficacy, showed effective and specific silencing (Fig. 5A), as evidenced by the significantly reduced HPRT-positive staining in the IF analysis (****p < 0.0001) (Fig. 5C). Both qRT-PCR (Fig. 4B) and IF analyses consistently demonstrate that DsiRNA2 and DsiRNA3 are the most effective at silencing *TGM7* (Fig. 5B), highlighting their potential as therapeutic candidates for targeting *TGM7* in testicular germ cell cancer. qRT-PCR results show significant reductions in *TGM7* mRNA levels with DsiRNA2 and DsiRNA3, while DsiRNA1 exhibits lower silencing efficacy.

Similarly, the IF results confirm these findings at the protein level, with marked reductions in TGM7-positive staining (Cy5) in cells treated with DsiRNA2 and DsiRNA3, as quantified by significant differences (****p < 0.0001) compared to the non-targeting siRNA control (Fig. 5C). DsiRNA1, however, shows a minimal effect on TGM7 protein expression, further emphasizing the variability in silencing efficiency among the tested DsiRNAs. Together, these results underscore the therapeutic potential of DsiRNA2 and DsiRNA3, highlighting the importance of HPRT as a reliable positive control, and demonstrating the value of selecting optimized siRNA sequences for effective gene silencing strategies. Although both DsiRNA2 and DsiRNA3 achieved significant knockdown at 24 h, DsiRNA3 was chosen for functional assays based on its superior early silencing efficiency, lower variability, and greater phenotypic impact in pilot studies. Additionally, while DsiRNA1 surpassed others by 48 h, its delayed onset made it less suitable for short-term assays such as MTT.

### 3.5 Dose-Dependent Inhibition of NT2/D1 Tumor Cell Proliferation by DsiRNA3 as Assessed by MTT Assay

**Figure 6:**
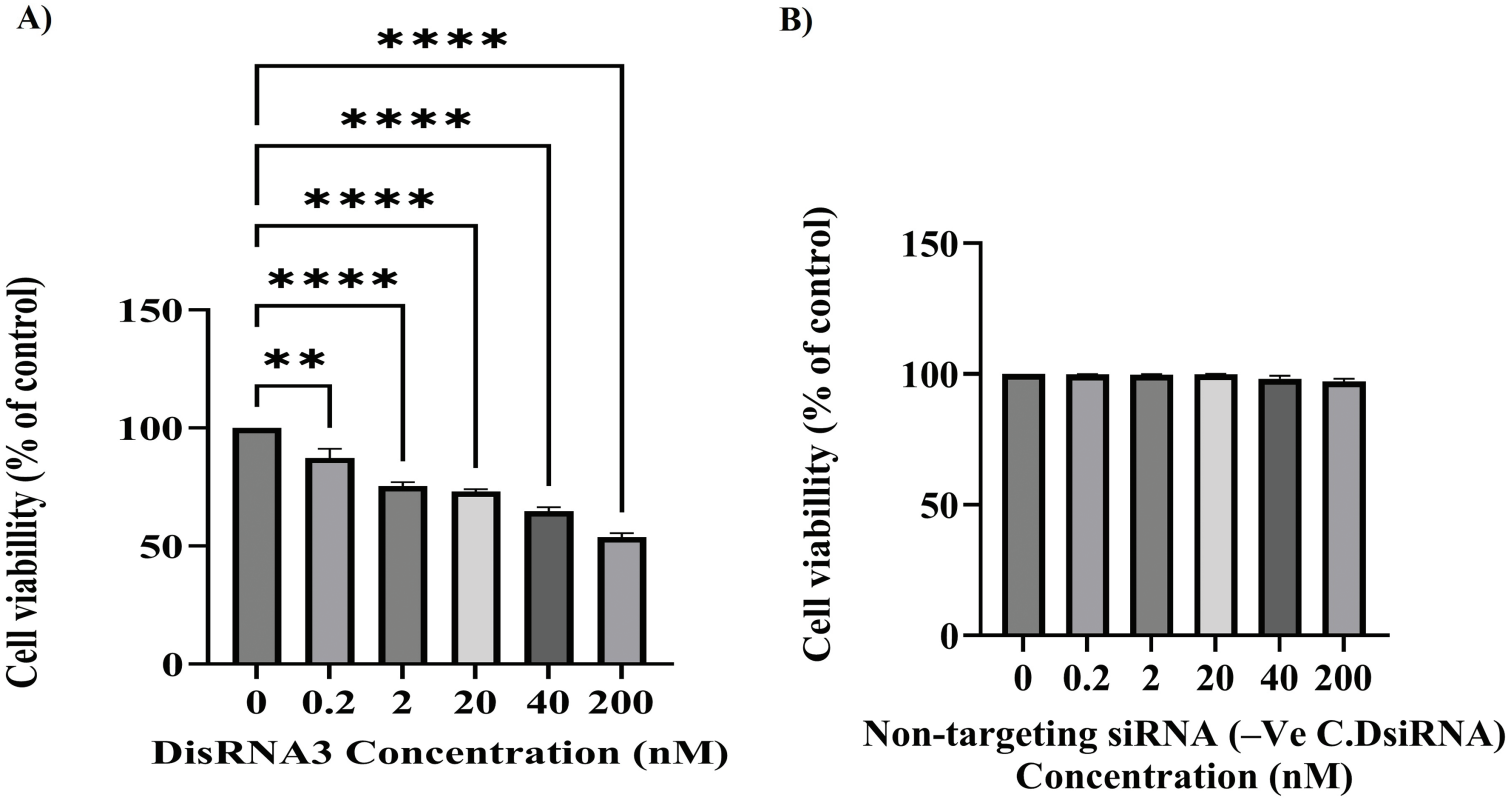
Dose-dependent inhibition of NT2/D1 tumor cell proliferation following TG7 silencing by DsiRNA3. NT2/D1 cells were transfected with increasing concentrations of DsiRNA3 targeting TGM7 (0, 0.2, 2, 20, 40, and 200 nM) (**A**) or with non-targeting siRNA (-Ve C.DsiRNA) (**B**) and incubated for 24 h. Cell viability was measured using the MTT assay. Results are presented as percentage viability relative to the untreated control (0 nM), which was set to 100%. The data represent mean ± SEM from two independent experiments performed in triplicate (*n* = 2 × 3). Statistical analysis was conducted using two-way ANOVA with Šídák’s multiple comparisons test. **p < 0.01, ****p < 0.0001

This experiment aimed to evaluate the ability of DsiRNA3, a TG7-targeting siRNA, to inhibit tumor cell proliferation in the NT2/D1 cell line across a range of concentrations. The MTT assay results (Fig. 6A) demonstrate that DsiRNA3 significantly inhibits the proliferation of NT2/D1 tumor cells in a clear dose-dependent manner. Cell viability, measured as a percentage relative to the control group (0 nM DsiRNA3), decreases progressively with increasing concentrations of DsiRNA3 (0.2, 2, 20, 40, and 200 nM). A modest reduction in cell viability is observed at the lowest concentration 0.2 nM (**p < 0.01), with more substantial inhibition at 2 and 20 nM (****p < 0.0001), and the most pronounced effect at 200 nM (****p < 0.0001), where viability was reduced by over 50%.

All concentrations tested showed statistically significant reductions in cell viability compared to the control group, as determined by two-way ANOVA followed by Šídák’s multiple comparisons test. These findings confirm the efficacy of DsiRNA3 in suppressing NT2/D1 tumor cell proliferation in a concentration-dependent manner and support its potential as a therapeutic agent targeting TG7. Although transfection optimization was conducted with up to 20 nM DsiRNA3 to limit cytotoxicity, the 200 nM concentration was used in the final viability assay (Fig. 6) to assess dose-dependent antiproliferative effects. To ensure the observed reduction in cell viability was not due to off-target effects, parallel experiments were conducted using non-targeting siRNA (-Ve C.DsiRNA) at equivalent concentrations. These control treatments showed no significant cytotoxicity compared to the 0 nM group, confirming the specificity of TG7 silencing by DsiRNA3 (Fig. 6B).

### 3.6 Gene Expression Modulation by DsiRNA3 in NT2/D1 Cells

**Figure 7:**
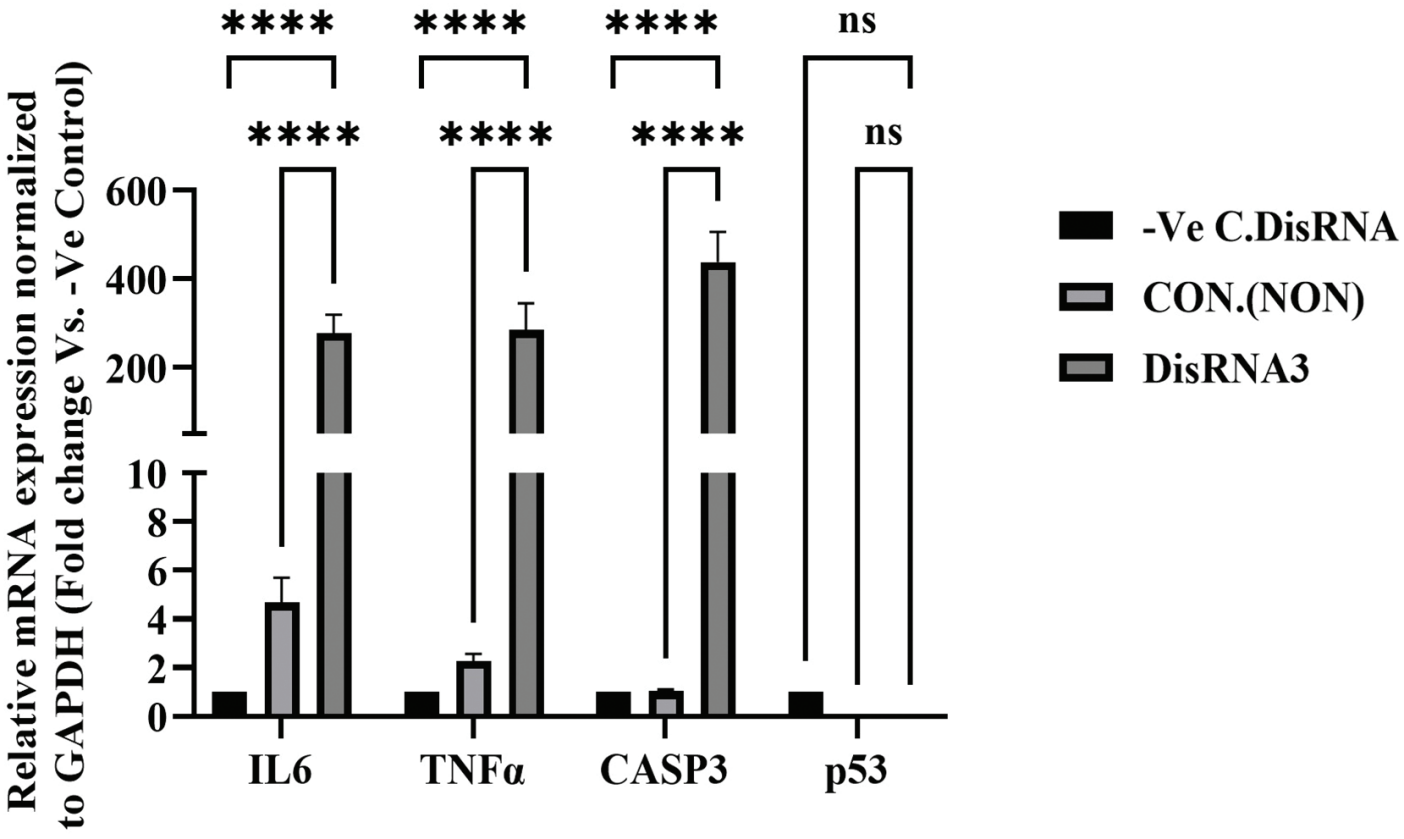
mRNA expression analysis of IL6, TNFα, CASP3, and p53 following TG7 silencing in NT2/D1 cells. Cells were treated with 20nM DsiRNA3, and expression was compared to non-treated controls (CON) and negative control siRNA (-Ve C). Expression levels were normalized to GAPDH and analyzed using qRT-PCR. Data is presented as fold-change relative to the -Ve control. The results represent mean ± SEM from three independent experiments (*n* = 3), each performed in triplicate. Statistical analysis was performed using One-way ANOVA with Šídák’s multiple comparisons test. ****p < 0.0001; ns, not significant

Pro-inflammatory cytokines and apoptosis biomarkers are likely to play an important role in human malignant testicular germ cell lines. In this study, we report the expression of TNFα, IL6, caspase-3, and p53 at the mRNA level in NT2/D1 following gene silencing of Transglutaminase 7 (TG7). As shown in Fig. 7, IL6 (Interleukin-6) expression was significantly (****p < 0.0001) increased in the DsiRNA-treated group compared to both the non-treated (Control Non) and negative control (-Ve C, DsiRNA) groups. This finding suggests that TGM7 silencing leads to the upregulation of IL6 expression.

Similar to IL6, TNFα (Tumor Necrosis Factor-alpha) expression was significantly higher (****p < 0.0001) in the DsiRNA-treated group, indicating an inflammatory response triggered by TGM7 gene silencing. The expression of CASP3, a key apoptosis biomarker, was also markedly elevated (****p < 0.0001) in the DsiRNA-treated group, suggesting enhanced apoptotic activity following TGM7 knockdown. In contrast, the expression of p53 showed no statistically significant change after TGM7 silencing, indicating that p53 is not a major effector in the siRNA-induced response under the tested conditions (Fig. 7). This suggests that TGM7’s role in regulating apoptosis in NT2/D1 cells may occur through a p53-independent pathway.

## 4 Discussion

Testicular germ cell tumors (TGCTs) are the most prevalent form of testicular malignancies, constituting the vast majority of cases and primarily affecting males in early adulthood [1–3]. Despite advancements in understanding the molecular underpinnings of testicular cancer, the role of transglutaminases (TGs), particularly TG7, has remained largely unexplored. This study provides novel insights into the molecular characterization of TGs in testicular cancer, focusing on TG7 gene expression, protein localization, and functional significance in tumor progression.

The elevated expression of TG7 in tumor nests and stromal areas points to a possible role in tumor stroma interaction, perhaps facilitating extracellular matrix remodeling and creating a pro-survival microenvironment. While TG2, another transglutaminase isozyme, has been shown to reshape the tumor niche by regulating key signaling pathways and tissue biomechanics [18], TG7 may function similarly, possibly influencing cell adhesion, proliferation, or immunomodulation. In testicular germ cell tumor models, TG7 silencing led to reduced viability and upregulation of IL6 and TNFα, suggesting that TG7 might suppress apoptotic or inflammatory signals under physiological conditions. This mechanism could resemble recent findings where TG2 modulation of IL6 contributes to immune suppression and tumor progression [30,31]. Together, these observations hint that TG7 may affect tumor progression through indirect regulation of pro-inflammatory and survival pathways, rather than classic apoptotic cascades, a hypothesis that requires further molecular investigation. HPRT was included as a positive control in siRNA transfection experiments due to its stable expression across various cell types. As a housekeeping gene encoding hypoxanthine-guanine phosphoribosyltransferase, HPRT plays a crucial role in purinergic signaling, influencing cellular responses to extracellular nucleotides [32,33]. Its involvement in tumor proliferation and metabolic regulation highlights its significance in cancer research [33]. HPRT confirmed transfection efficiency and gene silencing, reinforcing the findings on TG7’s role in tumor cell viability.

The observed differential expression of TG isoforms, particularly the marked elevation of TG7 in cancerous conditions, suggests a prominent role in regulating tumor cell survival, adhesion, and extracellular matrix interactions [18].

Immunohistochemical and immunofluorescence analyses revealed minimal TG7 expression in normal testes, primarily in Leydig cells and basal seminiferous tubules, suggesting a limited physiological role. Conversely, cancerous tissues exhibited strong TG7 expression in tumor nests and stromal regions, supporting its involvement in tumor proliferation, invasion, and stromal remodeling [34]. Although IHC and IF both revealed low TG7 expression in normal testicular tissue, slight differences in localization were noted. IHC showed sporadic expression in the basal compartment of seminiferous tubules and interstitial areas, while IF highlighted a stronger signal between tubules, suggesting Leydig cell localization. These discrepancies may arise from the enhanced spatial resolution and sensitivity of IF, which facilitates more precise subcellular localization than IHC’s chromogenic detection method [35]. Nonetheless, both techniques support the conclusion that TG7 is primarily expressed in non-spermatogenic cells within the testicular microenvironment.

TG7 knockdown experiments using DsiRNA duplexes confirmed its functional relevance in tumor biology, highlighting its potential as a molecular target for gene silencing strategies in TGCT treatment. Immunofluorescence confirmed reduced TG7 protein levels, highlighting TG7’s potential as a therapeutic target in testicular cancer [18]. These findings are consistent with the growing literature supporting RNAi-based cancer therapy strategies. Several studies have shown that siRNA-mediated knockdown of tumor-associated genes such as TGM2, EGFR, and MUC1 in solid tumors can effectively reduce tumor proliferation and enhance apoptotic pathways [14,17].

TG7 silencing significantly impaired NT2/D1 cell proliferation, reinforcing its potential role in tumor progression and supporting its candidacy as a therapeutic target. These findings are consistent with prior research in prostate and breast cancer models, where transglutaminase inhibition via DsiRNA strategies effectively suppressed tumor growth [36,37]. The upregulation of TG7 in testicular cancer suggests its involvement in tumorigenesis through apoptosis modulation and extracellular matrix remodeling [38]. The stromal localization of TG7 suggests interactions with the tumor microenvironment, potentially facilitating tumor progression and metastasis [39,40].

TGM7 silencing in NT2/D1 cells resulted in upregulated pro-inflammatory cytokines (IL6, TNFα) and apoptosis biomarker CASP3. Although p53 expression trended downward, the reduction was not statistically significant, suggesting a limited role for p53 under the tested conditions. These findings indicate that TGM7 knockdown-induced apoptosis is likely p53-independent, with caspase-3 activation and cytokine upregulation (IL6, TNFα) being more critical downstream effects. Similar p53-independent apoptotic pathways have been reported in various models, including Apollon-knockdown breast cancer cells, where apoptosis occurred primarily via caspase-3 activation without p53 involvement [41], and in genistein-treated HCT116 colon cancer cells lacking functional p53 [42]. Additionally, TNFα has been shown to induce apoptosis through NF-κB-mediated PUMA expression independently of p53 [43].

Transglutaminases, including TGM7, regulate inflammation [38]. Other TGs, such as TGM2, modulate IL6 expression in various cancers, promoting tumor progression [44]. Increased IL6 and TNFα expression in this study suggests that TGM7 silencing disrupts inflammatory regulation, creating a tumor-supportive microenvironment [45]. Similar cytokine responses have been reported following TGM2 knockdown in colorectal and breast cancer models, indicating that TG modulation broadly influences tumor immune responses [16,18].

Upregulated CASP3 following TGM7 silencing suggests an anti-apoptotic role for TGM7 in NT2/D1 cells. TGM2 is known to promote survival by inhibiting caspase activation [46]. The simultaneous increase in IL6 and TNFα could indicate a dual role for TGM7 in modulating inflammatory and apoptotic pathways [47]. While TG7’s role in testicular germ cell tumors has been largely unexplored, transglutaminases are increasingly linked to cancer progression, metastasis, and chemoresistance [48]. The cytokine upregulation following TGM7 silencing mirrors findings in colorectal and breast cancer research, where TG modulation alters cytokine expression [49].

Insights from TGM2 research suggest potential interactions between transglutaminases and caspase-3 during apoptosis [50,51]. Moreover, the observed dissociation between p53 and caspase-3 responses in our model may indicate the activation of a non-canonical apoptotic pathway following TG7 inhibition, similar to the findings in siRNA-treated, p53-deficient tumor cells [15]. The upregulation of CASP3 following TG7 silencing could reflect a feedback mechanism promoting apoptosis, and this warrants further mechanistic investigation. While p53 plays a central role in regulating apoptosis and cell cycle progression [52] and TGs such as TGM2 have been shown to modulate p53 activity [53], our results suggest TG7 may regulate apoptosis through alternative p53-independent mechanisms. While this study did not employ CRISPR-Cas9 validation, future investigations will use CRISPR-based gene editing to confirm target specificity and rule out potential off-target effects of DsiRNA-mediated TG7 silencing.

Beyond TG7’s role in tumor cell proliferation, recent literature suggests that transglutaminases contribute more broadly to tumor biology. For instance, TG2 has been implicated in angiogenesis, immune evasion, and resistance to therapy through crosslinking of extracellular matrix proteins and modulation of survival pathways [18]. These functions may extend to TG7, which shares conserved domains and possibly overlapping regulatory mechanisms. Furthermore, TGs are increasingly recognized for their influence on cancer stemness and epigenetic plasticity, contributing to immune resistance and tumor heterogeneity [54]. A recent pharmacological review also highlighted the potential of targeting TGs as part of integrative drug discovery pipelines using advanced modeling and bioinformatics tools [55]. In addition, angiogenesis pathways, which are known to be regulated by TG2, could be indirectly influenced by TG7, suggesting a potential role in the vascular remodeling observed in testicular tumors [56]. These emerging insights underscore the importance of further mechanistic studies on TG7’s contribution to tumor plasticity, microenvironmental crosstalk, and therapy resistance.

Although our study establishes a foundational role for TG7 in modulating inflammatory and apoptotic signaling in TGCT cells, the precise downstream pathways remain undefined. Future investigations will include Western blot analyses of key signaling mediators (e.g., phosphorylated NF-κB, STAT3, and cleaved caspase-3) and chromatin immunoprecipitation (ChIP) assays to identify TG7-associated genomic targets. These approaches will be essential to elucidate how TG7 regulates gene expression and contributes to testicular tumor biology at the molecular level. In conclusion, this study provides the first evidence of TG7’s overexpression and functional role in testicular germ cell tumor progression. Gene silencing using DsiRNA3 not only suppressed cell viability but also disrupted key inflammatory and apoptotic regulators. These findings support TG7 as a novel therapeutic target and underscore the value of siRNA-based strategies in TGCT management.

Although no selective TG7-specific inhibitors are currently available, structural similarities across the transglutaminase family provide a foundation for rational drug design. For example, Sulforaphane (SFN), an irreversible TG2 inhibitor, stabilizes the enzyme in an open, inactive conformation and has demonstrated anticancer efficacy in several models [18]. Similarly, a small-molecule inhibitor (TG53) disrupts TG2-fibronectin interactions, inhibiting cell migration and invasion [57]. Moreover, studies using cell-impermeable TG2 inhibitors have confirmed that intracellular TG2 drives pro-oncogenic phenotypes in various solid tumors, supporting intracellular targeting strategies [12]. These findings suggest that TG2-targeted scaffolds could potentially be adapted for TG7 inhibition.

While the specific active-site characteristics of TG7 remain less defined, phage display peptide screening has revealed substrate preferences, providing a framework for rational inhibitor design and virtual screening [58]. Chemical probe databases such as ChEMBL and BindingDB may be explored to identify TG2-based scaffolds suitable for TG7. Additionally, *in silico* docking using homology-modeled TG7 structures could help identify novel lead compounds. As structural knowledge of TG7 and its substrate specificity expands, such small molecules could complement RNAi-based approaches and offer additional strategies for managing TGCTs.

Despite promising leads based on TG2 inhibitor scaffolds, the development of highly specific TG7 inhibitors remains challenging due to the conserved catalytic domains and substrate-binding pockets shared across transglutaminase isoforms. These structural similarities increase the risk of cross-reactivity and off-target effects. Selectivity will likely require access to high-resolution TG7 structural data and advanced *in silico* screening to exploit isoform-specific conformational features [57,59].

*Limitations of the Study*

This study has several limitations. First, the absence of Western blotting limited our ability to confirm TG7 silencing and downstream protein expression at the protein level. Second, apoptosis was inferred through mRNA expression (CASP3), which may not fully capture cellular outcomes; thus, future studies should include functional assays such as Annexin V/PI staining and cleaved caspase-3 analysis. Lastly, while IF and IHC analyses provide localization insights, quantitative analysis of TG7 protein expression was not performed due to a lack of access to suitable digital pathology software.

## 5 Conclusion

This study provides novel insights into the role of transglutaminase 7 (TG7) in testicular germ cell tumor (TGCT) biology. We demonstrated that TG7 is significantly upregulated in tumor tissues compared to normal testicular samples, with pronounced expression observed in tumor nests and stromal regions. Functional analyses confirmed that TG7 promotes tumor cell proliferation, and that its silencing using DsiRNA3 led to a dose-dependent reduction in cell viability, accompanied by the upregulation of pro-inflammatory cytokines IL6 and TNFα, as well as the apoptotic marker CASP3. Although p53 expression was also reduced following TG7 knockdown, the change was not statistically significant, suggesting that TG7 may modulate apoptosis through p53-independent mechanisms.

The inclusion of HPRT1 as a positive control further validated the siRNA transfection approach and ensured the reliability of the gene silencing results. Collectively, these findings establish TG7 as a key regulator of tumor cell viability and immune response modulation, reinforcing its potential utility as both a biomarker and therapeutic target in TGCT.

Future research should aim to elucidate the downstream signaling mechanisms mediated by TG7 through the protein-level analysis of transcriptional and apoptotic regulators, including NF-κB, STAT3, and caspase-3, as well as through chromatin immunoprecipitation assays to determine its genomic targets. Additionally, investigating TG7’s involvement in tumor-stroma interactions, metastasis, and its association with clinical outcomes will be essential to advancing its translational potential. The development of TG7-targeted therapeutics, including siRNA-based approaches and small-molecule inhibitors, could contribute to more effective and personalized strategies for managing testicular cancer.

## Data Availability

Data available within the article.
